# Extensions of the probabilistic ranking metrics of competing treatments in network meta‐analysis to reflect clinically important relative differences on many outcomes

**DOI:** 10.1002/bimj.201900026

**Published:** 2019-10-29

**Authors:** Dimitris Mavridis, Raphaël Porcher, Adriani Nikolakopoulou, Georgia Salanti, Philippe Ravaud

**Affiliations:** ^1^ Department of Primary Education University of Ioannina Ioannina Greece; ^2^ Faculté de Médecine Université Paris Descartes Paris France; ^3^ Institute of Social and Preventive Medicine (ISPM) University of Bern Bern Switzerland; ^4^ Department of Epidemiology Columbia University Mailman School of Public Health New York USA

**Keywords:** benefit–risk assessment, clinically important value, multiple outcomes, network meta‐analysis, ranking metrics

## Abstract

One of the key features of network meta‐analysis is ranking of interventions according to outcomes of interest. Ranking metrics are prone to misinterpretation because of two limitations associated with the current ranking methods. First, differences in relative treatment effects might not be clinically important and this is not reflected in the ranking metrics. Second, there are no established methods to include several health outcomes in the ranking assessments. To address these two issues, we extended the P‐score method to allow for multiple outcomes and modified it to measure the mean extent of certainty that a treatment is better than the competing treatments by a certain amount, for example, the minimum clinical important difference. We suggest to present the tradeoff between beneficial and harmful outcomes allowing stakeholders to consider how much adverse effect they are willing to tolerate for specific gains in efficacy. We used a published network of 212 trials comparing 15 antipsychotics and placebo using a random effects network meta‐analysis model, focusing on three outcomes; reduction in symptoms of schizophrenia in a standardized scale, all‐cause discontinuation, and weight gain.

## 1 INTRODUCTION

Systematic reviews increasingly employ network meta‐analysis (NMA) to compare three or more competing interventions for a condition (Petropoulou et al., 2017). The two main outputs of a NMA are the relative treatment effects between all treatments and treatment ranking. The former is usually presented in forest plots of summary effects (e.g., all treatments vs. the reference one) or in a league table that includes all relative effects. This is easily achieved with the *netleague* command in R (RStudio Team, 2015) and Stata (Chaimani, Higgins, Mavridis, Spyridonos, & Salanti, 2013; Rücker, Schwarzer, Krahn, & König, 2018; StataCorp, 2013). At the same time, several graphical and quantitative measures have been developed for ranking interventions (Rücker & Schwarzer, 2015; Salanti, Ades, & Ioannidis, 2011). Many of the measures use the distribution of relative effects to estimate probabilities for any intervention assuming any possible rank. Such probabilities are easily estimated in popular software for NMA (Lunn, Thomas, Best, & Spiegelhalter, 2000; Rücker et al., 2018; White, 2015). The most commonly used ranking approaches include the probability of each treatment to produce the best outcome (Pbest), rankograms, mean rank, estimating the surface under the cumulative ranking curve (SUCRA) and their equivalent P‐scores (Rücker & Schwarzer, 2015; Salanti et al., 2011; Trinquart, Attiche, Bafeta, Porcher, & Ravaud, 2016). PRISMA guidelines state that ranking metrics can be reported along with corresponding estimates of pairwise comparisons between interventions as they may exaggerate small differences in relative effects if looked at in isolation (Hutton et al., 2015).

The Pbest was largely employed when NMA was first introduced and up until 2016, 43% of NMAs providing a treatment hierarchy were using that metric for ranking interventions (Petropoulou et al., 2017). However, this probability ignores the entire distribution of rank probabilities and places emphasis only on one end of the distribution. Some interventions may be studied in a couple of small studies and their effects cannot be informed precisely by the network. As a result, they end up having the same probability for any possible rank, for example, if there are four interventions, one may have 25% probability of assuming any rank from 1 up to 4. Then, the probability of the intervention to rank first is quite high (25%) but so is the probability to rank last.

We can explore the entire ranking distribution by creating rankograms that depict the probability for any intervention of assuming any possible rank. Ranking distributions can be produced by resampling from the posterior relative treatment effects distributions (if NMA is fitted in a Bayesian environment) or by simulating from the estimated effects and their variance–covariance matrix (if NMA is fitted in a frequentist setting). If there is much uncertainty associated with an intervention, this will be reflected in a flat ranking distribution (Salanti, Del Giovane, Chaimani, Caldwell, & Higgins, 2014). Salanti et al. suggested summarizing the ranking distribution by calculating the surface under the cumulative ranking curve (SUCRA). Chaimani et al. (2013) suggested using multidimensional scaling techniques to visualize the level of similarity in the ranking between interventions. Rücker and Schwarzer (2015) developed a measure called P‐scores, which is equivalent to SUCRA but is not simulation‐based and can be computed analytically.

Most meta‐analyses report on many outcomes and typically each outcome is analyzed and ranked separately. In practice, treating physicians and patients making decisions weigh the benefits and risks of each intervention and such a decision is difficult to communicate without a systematic method on how to conduct a benefit–risk assessment. Outcomes both within and across studies are correlated. Ideally, we would like to analyze outcomes in a single framework by using multivariate meta‐analysis (Mavridis & Salanti, 2013) and multiple outcome network meta‐analysis (Efthimiou et al., 2014, 2015). Methods to rank interventions taking into account multiple outcomes have been suggested in the literature. A simple approach is to combine ranking measures for two different outcomes in a single plot (e.g., a scatterplot of the SUCRA/P‐scores value for one outcome vs. the SUCRA/P‐scores value for another outcome or rankograms for different outcomes presented in the same figure). Veroniki, Straus, Fyraridis, and Tricco (2016) presented the rank–heat plot, a simple graphical approach to present treatment ranking including multiple outcomes. Tervonen et al. (2015) provide guidance on applying multiple criteria decision analysis in benefit–risk assessment. Rücker and Schwarzer (2017) suggest using partial ordering to reveal both orders of treatments that hold for all outcomes and sets of treatments where ordering is not the same across all outcomes.

Primary and secondary outcomes are not equally important and there is usually no consensus on their importance as different stakeholders (e.g., clinicians, patients) have different perspectives. Naci, van Valkenhoef, Higgins, Fleurence, and Ades (2014) suggest that individual stakeholders can assign weights to all outcomes so that we result in individual rankings. Another approach that focuses on patients preferences is based on the concept of a Minimal Clinically Important Difference (MCID), which determines the smallest amount an outcome must change to be meaningful to patients (Jaeschke, Singer, & Guyatt, 1989). Several methods have been suggested for determining the MCID (Johnston et al., 2015; Rai, Yazdany, Fortin, & Aviña‐Zubieta, 2015).

In this paper, we use the minimum Clinically Important Value (CIV) for a relative effect for each outcome. Conditional on the minimum CIV, we may produce a ranking of interventions. Suppose that we have two interventions A and B and two outcomes, one for efficacy and one for safety, with intervention A being more efficacious but less safe. We aim to extend the ranking metrics to address situations where multiple outcomes are of interest and differentiate between clinically important and unimportant treatment effects. Conditioning on the CIVs, we get a ranking that is based on how much one is willing to tolerate for specific gains. We advocate the use of graphical measures and show how P‐scores can be presented graphically for a benefit–risk assessment. A similar approach has been suggested that focuses on Pbest (Brignardello‐Petersen, Johnston, Jadad, & Tomlinson, 2018). In Section 2, we present the ranking metrics for one and multiple outcomes as well as how P‐scores can be used for a benefit–risk assessment using CIVs. In Section 3, we illustrate the methods presented in Section 2 using a network of 212 randomized controlled trials (RCTs) comparing antipsychotics. We conclude in Section 4 with a discussion.

## 2 RANKING METRICS

### 2.1 Ranking metrics for a single outcome

Suppose that we have *I* interventions and $P_{ir}$ refers to the probability that intervention *i* assumes rank *r* with i,r=1,…,I. These rank probabilities form a discrete distribution $\sum_{r=1}^{I}P_{ir}=1$ with cumulative distribution function (cdf) $F\left(i,x\right)=\sum_{r=1}^{x}P_{ir}$. Salanti et al. (2011) suggested a summary ranking metric, called SUCRA, that is based on summarizing the surface under the cumulative ranking curve using a step function. An ideal intervention would have SUCRA = 1 because it would have probability 1 to achieve the top rank place and zero probability of achieving any other place ($P_{i1}=1$ and $P_{ir}=0\;\forall \;r\neq 1$). A SUCRA value for an intervention *i* is the proportion of competing treatments worse than *i* (Rücker & Schwarzer, 2015) and is computed as

$$\mathrm{SUCR}A_{i}=\frac{1}{I-1}\sum_{r=1}^{I-1}F\left(i,r\right)=\frac{1}{I-1}\sum_{r=1}^{I-1}\sum_{x=1}^{r}P_{ix}$$

and it has a one‐to‐one relationship with the average or mean rank $\left(E(\mathrm{ran}k_{i})\right)$. More specifically,

$$\mathrm{SUCR}A_{i}=\frac{I-E\left(\mathrm{ran}k_{i}\right)}{I-1}.$$

In meta‐analysis models, normal distributions are conventionally assumed for the absolute and, subsequently, for the relative effects. More specifically, we assume that $\mu_{i}∼N\left({\hat{\mu }}_{i},s_{i}^{2}\right)\;\forall \;i$ where $\mu_{i}$ is the effect of treatment *i* on the health outcome estimated with variance $s_{i}^{2}$. For any pair of interventions *i* and *j* we have

(1) $$P_{i>j}=P\;\left(\mu_{i}>\mu_{j}\right)=\Phi \left(\frac{{\hat{\mu }}_{i}-{\hat{\mu }}_{j}}{s_{ij}}\right),$$

where $P_{i>j}$ is interpreted as the extent of certainty that the outcome for *i* treatment, $\mu_{i}$, is larger than $\mu_{j}$ and Φ is the cdf of a standard normal distribution. Note that when using ratios as effect sizes (odds/risk ratio) Equation (1) should have the difference in outcomes on the logarithmic scale. When the outcome is harmful, *i* is preferable than *j* when $\mu_{i}<\mu_{j}$.

The difference ${\hat{\mu }}_{i}-{\hat{\mu }}_{j}$ and its standard error $s_{ij}$ are standard outputs of a NMA and they are typically reported in a league table. Generally, there are $\left(\begin{array}{c} I\\ 2 \end{array}\right)$ effect sizes (${\hat{\mu }}_{i}-{\hat{\mu }}_{j})$ and 95% confidence/credible intervals (or alternatively standard errors $s_{ij}$). Rücker and Schwarzer (2015) consider the mean value

(2) $${\bar{P}}_{i}=\frac{1}{\left(I-1\right)}\sum_{\left(r,r\neq i\right)}^{I}P_{i>r},$$

where ${\bar{P}}_{i}$ is interpreted as the mean extent of certainty that $\mu_{i}$ is larger than any other $\mu_{j}$, averaged over all competing interventions *j* (j≠i) with equal weights. Rücker and Schwarzer (2015) named P‐score the summary measure estimated in Equation (2) and provided a formal proof that P‐scores and SUCRA values are identical if the true probabilities are known.

### 2.2 Ranking metrics for multiple outcomes

Suppose that we have two interventions *i* and *j* and two outcomes *O*_1_ and *O*_2_. Without loss in generality we suppose that for both outcome the larger the value the better the treatment. The probability that intervention *i* is better than intervention *j* in both outcomes *O*_1_ and *O*_2_ is

$$\begin{array}{ccc} P_{i>j} & = & P\left(\mu_{i_{O_{1}}}>\mu_{j_{O_{1}}}\cap \mu_{i_{O_{2}}}>\mu_{j_{O_{2}}}\right)\\ & = & P\left(Z_{O_{1}}<\frac{{\hat{\mu }}_{i_{O_{1}}}-{\hat{\mu }}_{j_{O_{1}}}}{\sigma_{ij_{O_{1}}}}\;\cap Z_{O_{2}}<\frac{{\hat{\mu }}_{i_{O_{2}}}-{\hat{\mu }}_{j_{O_{2}}}}{\sigma_{ij_{O_{2}}}}\right)=\Phi_{2}\;\left(Z_{O_{1}}<\frac{{\hat{\mu }}_{i_{O_{1}}}-{\hat{\mu }}_{j_{O_{1}}}}{\sigma_{ij_{O_{1}}}},Z_{O_{2}}<\frac{{\hat{\mu }}_{i_{O_{2}}}-{\hat{\mu }}_{j_{O_{2}}}}{\sigma_{ij_{O_{2}}}}\right), \end{array}$$

where $Z_{O_{1}},Z_{O_{2}}∼N\left(0,1\right)$ and Φ_2_ is the cdf of a standard bivariate normal distribution with between‐study correlation $\rho_{O_{1}O_{2}}^{\mathrm{between}}$. We used R package mvtnorm (Hothorn, Bretz, & Genz, 2001) to compute cumulative probabilities from a multivariate normal distribution.

Ideally, a multivariate NMA would provide us with correlation estimates but the method is not so easy to use in practice. Two common problems in multivariate meta‐analysis are that the within‐study correlations are usually not reported in individual studies and the between‐study correlation is poorly estimated (Mavridis & Salanti, 2013).

The following strategies are possible:

1. Ignore the correlation and analyze each outcome separately ($\rho_{O_{1}O_{2}}^{\mathrm{between}}=0$)
2. Estimate the between‐study correlation using multivariate network meta‐analysis (Efthimiou et al., 2014, 2015) if within‐study correlations are known or can be assumed known.
3. Use expert opinion to inform $\rho_{O_{1}O_{2}}^{\mathrm{between}}$
4. Undertake a sensitivity analysis assuming a plausible range of values for $\rho_{O_{1}O_{2}}^{\mathrm{between}}$

The probabilities estimated in Equation (1) refer to one intervention producing values for an outcome that are preferable to those produced by the other treatment. Small differences are not necessarily clinically important and may be irrelevant.

Suppose that we are interested in the probability that the relative effect of *i* versus *j* is greater than a Clinically Important Difference ($\mathrm{CI}V^{ij}$).

We modify Equation (1) to be

$$P_{i>j}=P\left(\mu_{i}-\mu_{j}-\mathrm{CI}V^{ij}>0\right)=\Phi \left(\frac{{\hat{\mu }}_{i}-{\hat{\mu }}_{j}-\mathrm{CI}V^{ij}}{s_{ij_{O_{1}}}}\right)$$

and then use these probabilities to estimate P‐scores using Equation (2).

This new modified P‐score is a CIV ranking metric and it reflects the mean extent of certainty that $\mu_{i}$ is larger than any other $\mu_{j}+\mathrm{CI}V^{ij}$ averaged over all competing interventions. For brevity, we assume that the CIV is the same for all treatment comparisons *i* versus *j* $\left(\mathrm{CI}V^{ij}=\mathrm{CIV}\right)$. Ideally, we would like CIV to be informed by a method that reflects patient perceptions. If its value is unknown, we may consider a range of values for CIV.

### 2.3 Benefit–risk assessment using P‐scores and CIVs

In many cases, it is the most effective drugs that perform poorly in terms of adverse events. Decision‐makers need to assess the benefit/risk of profile for competing interventions. Although subjectivity in the assessment of the benefit–risk profile is unavoidable, there have been attempts to formalize the process for evaluating the balance between healthcare interventions (Najafzadeh et al., 2015; Puhan, Singh, Weiss, Varadhan, & Boyd, 2012; Tervonen et al., 2015; van Valkenhoef et al., 2012). Most methods require weighting the various outcomes either by patients or from other sources (i.e., clinicians) and then, benefit–risk methods estimate some function of the weights and the probability of experiencing a beneficial and a harmful outcome. We present below a visual method that shows how ranking changes by using different CIV values for harmful and beneficial outcomes.

We consider differences in summary estimates smaller or larger than certain effects (the CIVs). For example, we may want to estimate the probability that *i* is better than*j* in efficacy (outcome *O*_1_) by a certain amount CIV_1_ when their differences in acceptability (outcome *O*_2_) is less than CIV_2_.

$$P\left(\mu_{i_{O_{1}}}-\mu_{j_{O_{1}}}-\mathrm{CI}V_{1}>0\cap \mu_{i_{O_{2}}}-\mu_{j_{O_{2}}}-\mathrm{CI}V_{2}>0\right).$$

By changing CIV_1_ and CIV_2_, we can see how this probability fluctuates and using Equation (2), we can take a graphical presentation of the P‐scores that will give us the ranking of treatments for various pairs of CIV_1_ and CIV_2_.

Similarly, we may expand the method to more outcomes by adding extra parameters. Such computations will allow us to consider various tradeoffs between efficacy and acceptability and conduct a risk‐benefit assessment. We provide easy‐to‐use R code that handles all cases presented in this section (https://github.com/DimitrisMavridis/RankingNMA/blob/master/extendedP-scores).

## 3 APPLICATION: RANKING ANTIPSYCHOTICS FOR SCHIZOPHRENIA

### 3.1 Description of the dataset

We use a network of 212 randomized controlled trials (RCTs) and 43,049 participants comparing 15 antipsychotic drugs and placebo (Leucht et al., 2013). Details about the methodology and results for this systematic review can be found in the relevant publication (Leucht et al., 2013). In this manuscript, we focused on the primary efficacy outcome measured by the overall change in symptoms on a validated scale, all‐cause discontinuation (acceptability) and weight gain. Efficacy and weight gain are continuous outcomes and treatment differences are measured with the Standardized Mean Difference (SMD) whereas the Odds Ratio (OR) is used for acceptability. We transform the ORs to SMDs using formula $\mathrm{SMD}=\frac{\sqrt{3}}{\pi }\;\log\mathrm{OR}$ (Chinn, 2000).

### 3.2 Ranking antipsychotics for one outcome

**Figure 1:**
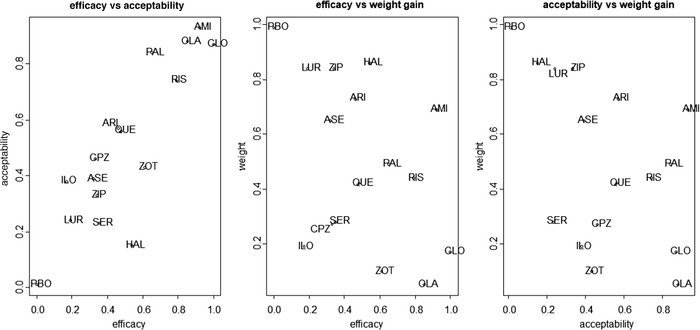
Three two‐way scatter plots for the SUCRA values for each of the pairs of the following outcomes (efficacy, acceptability, weight gain)

Table 1 shows the P‐scores and the ranks for each outcome. Figure 1 shows the scatterplots for the SUCRA values for each of the pairs of the following outcomes; efficacy, acceptability and weight. If we focus on reduction in symptoms (efficacy) and all‐cause discontinuation (acceptability), we see that clozapine, amisulpride, olanzapine, risperidone, and paliperidone form a distinct class of drugs taking the five top ranks in both outcomes. It is also noteworthy that although haloperidol performs pretty satisfactorily on efficacy (7th rank), it performs poorly on acceptability (15th rank). If we include weight gain, ranking is not straightforward. Clozapine and olanzapine perform poorly on weight gain and naturally, placebo ranks top in the hierarchy for this outcome. Only amisulpride performs well in all three outcomes. Clozapine and olanzapine perform very good in the lower (efficacy–acceptability) plane.

**Table 1:** P‐scores (as percentages %) and rank for each antipsychotic and three outcomes as obtained from three independent network meta‐analyses models

	Efficacy		All‐cause discontinuation		Weight gain	
Antipsychotic	P‐scores (%)	Rank	P‐scores (%)	Rank	P‐scores (%)	Rank
Clozapine (CLO)	99	1	85	3	17	14
Amisulpride (AMI)	92	2	93	1	69	6
Olanzapine (OLA)	85	3	89	2	5	16
Risperidone (RIS)	79	4	73	5	44	9
Paliperidone (PAL)	64	5	85	4	49	8
Zotepine (ZOT)	61	6	40	9	10	15
Haloperidol (HAL)	53	7	15	15	86	2
Quetiapine (QUE)	49	8	56	7	42	10
Aripiprazole (ARI)	46	9	56	6	72	5
Sertaline (SER)	35	10	23	14	28	11
Ziprasidone (ZIP)	35	11	33	12	84	3
Chlorpromazine (CHL)	33	12	47	8	27	12
Asenapine (ASE)	32	13	40	10	65	7
Lurasidone (LUR)	19	14	25	13	84	4
Iloperidone (ILO)	17	15	40	11	19	13
Placebo (PLA)	0	16	1	16	99	1

**Figure 2:**
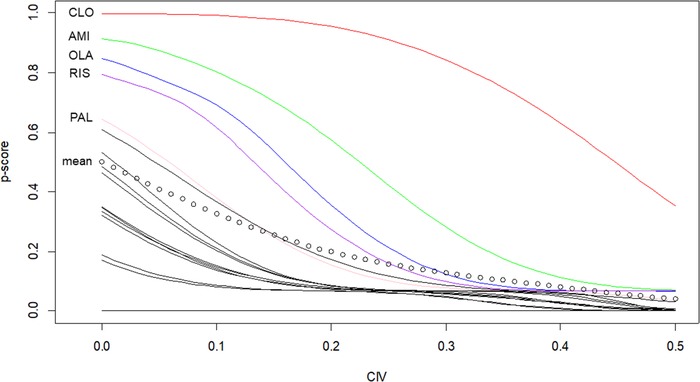
P‐scores for the 16 antipsychotics in terms of efficacy only, for various benefit (CIV) considerations. We have labeled only the lines with the most effective antipsychotics and placebo for illustration purposes. A dotted line is used to show the mean P‐score

Figure 2 shows how P‐scores for efficacy reduce for all antipsychotics for an increasing CIV (measured as the effect sizes on an SMD scale). For CIV = 0, we get the P‐scores in Table 2. In this case, the average P‐score across all antipsychotics is 0.5 but it is reduced for increasing CIV. We have labeled only the lines with the most effective antipsychotics and placebo for illustration purposes. The remaining lines drop very quickly, an indication that they are not much superior to placebo and, given the potential side‐effects of an active drug, one may not be willing to get a drug with a small effect. In those drugs, although there were some differences in the P‐scores for efficacy in the primary analysis (Table 2), differences become negligible as soon as we are looking for SMDs that differ by CIV = 0.1 units or more. It is also clear that P‐score for clozapine has a lower rate of decrease and remains the best choice even for large values of CIV. We also see that for CIV = 0.3 only amisulpride and clozapine are above the average P‐score.

**Table 2:** P‐scores (as percentages) for more than one outcome

Antipsychotic	Efficacy and acceptability	Efficacy, acceptability, and weight gain
Clozapine	85	14
Amisulpride	87	56
Olanzapine	81	3
Risperidone	70	26
Paliperidone	63	25
Zotepine	31	1
Haloperidol	13	5
Quetiapine	40	13
Aripiprazole	38	20
Sertaline	14	2
Ziprasidone	19	10
Chlorpromazine	24	4
Asenapine	21	8
Lurasidone	10	3
Iloperidone	13	1
Placebo	0	0

### 3.3 Ranking antipsychotics for several outcomes

For illustration purposes we set the correlation between efficacy and weight gain and acceptability and weight gain equal to −0.5 and the correlation between efficacy and acceptability equal to 0.5. Table 2 shows the extended P‐scores that take into account both efficacy and acceptability (second column) or all three outcomes (third column). P‐scores reduce for increasing number of outcomes, this is because we ask for interventions to be better than others in all three outcomes. When we move from analyzing only efficacy (Table 2) to analyzing both efficacy and acceptability, there is a small reduction in P‐scores. This is happening because efficacy and acceptability are positively correlated (Table 2). It seems that participants dropout from treatments that are ineffective. There is a sharp decrease in P‐scores in two of the most effective antipsychotics (clozapine and olanzapine) when we consider weight gain together with efficacy and acceptability (Table 2).

When all outcomes are considered (Table 2), amisulpride is ranked top (56%) with risperidone and paliperidone following on the second place (26%) while the most effective drug (clozapine) is ranked very low (14%) because it performs poorly in weight gain (Table 1 and Figure 1). For the benefit–risk assessment, we focus only on efficacy (*O*_1_) and weight gain (*O*_2_) for illustration purposes.

We considered a risk‐benefit assessment in which we are willing to tolerate a certain increase in the weight for a certain benefit in efficacy.

**Figure 3:**
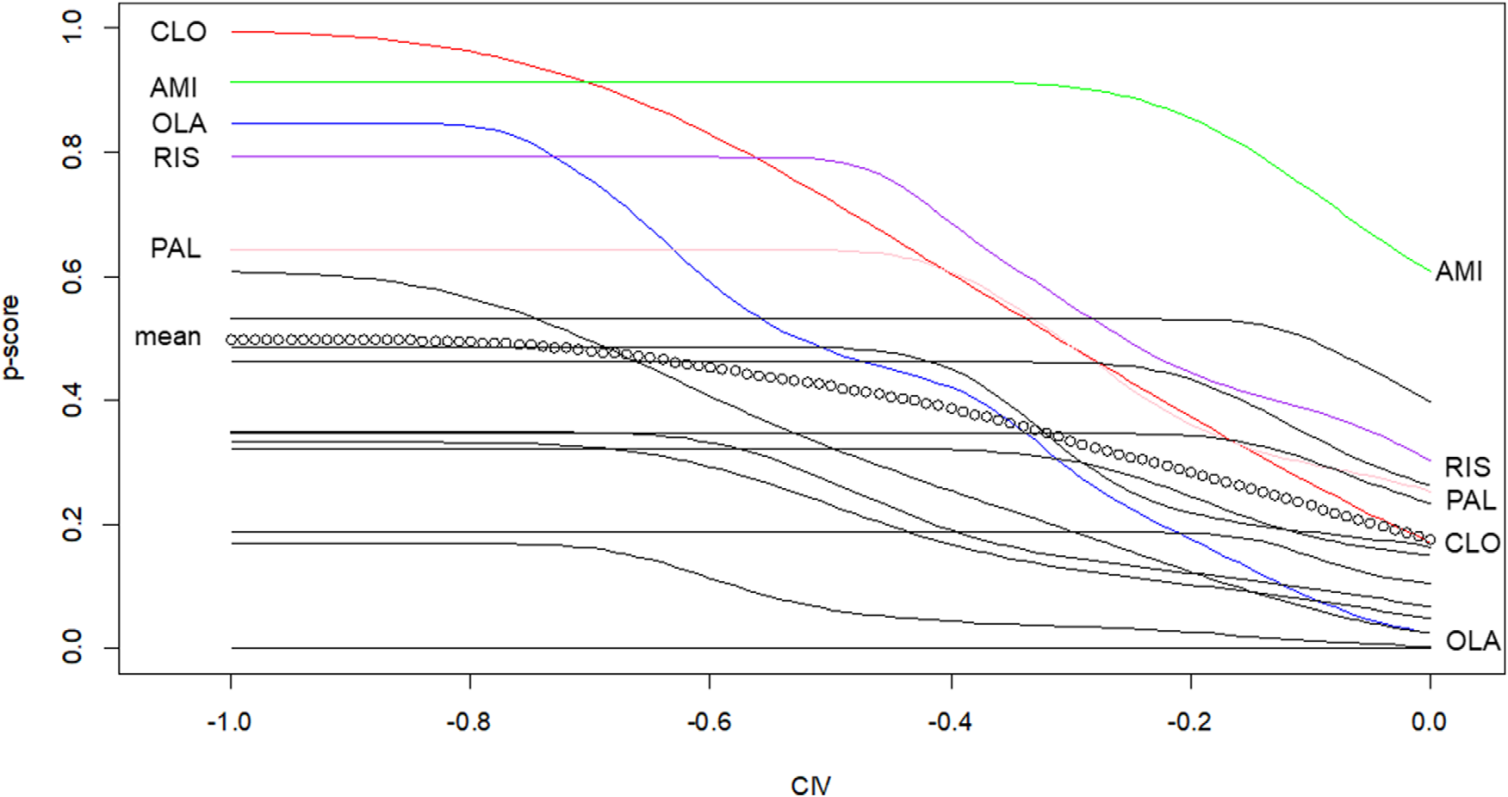
P‐scores for the 16 antipsychotics in terms of efficacy and weight, for various risk (CIV) considerations. We have labeled only the lines with the most effective antipsychotics for illustration purposes. A dotted line is used to show the mean P‐score

In Figure 3 we present P‐scores based on the probability $P(\left(\mu_{i_{O_{1}}}-\mu_{j_{O_{1}}}<0\right)\cap \left(\mu_{i_{O_{2}}}-\mu_{j_{O_{2}}}-\mathrm{CIV}<0\right))$ where CIV is the difference in weight gain we are willing to tolerate and it takes negative values. It varies between 0 and −1. SMDs of 0.6 are considered quite large and considering inference on these scenarios imply that retaining the baseline weight is not highly valued. Note that small values are desirable in both outcomes. Figure 3 shows that amisulpride is ranked high even for zero tolerance to weight gain. As we increase the amount of weight gain, we are willing to tolerate, clozapine, and olanzapine increase their P‐score. However, clozapine's P‐score exceeds amisulpride's when CIV=−0.65 and even exceeds the P‐scores of risperidone at CIV=−0.6 and paliperidone at CIV=−0.25.

**Figure 4:**
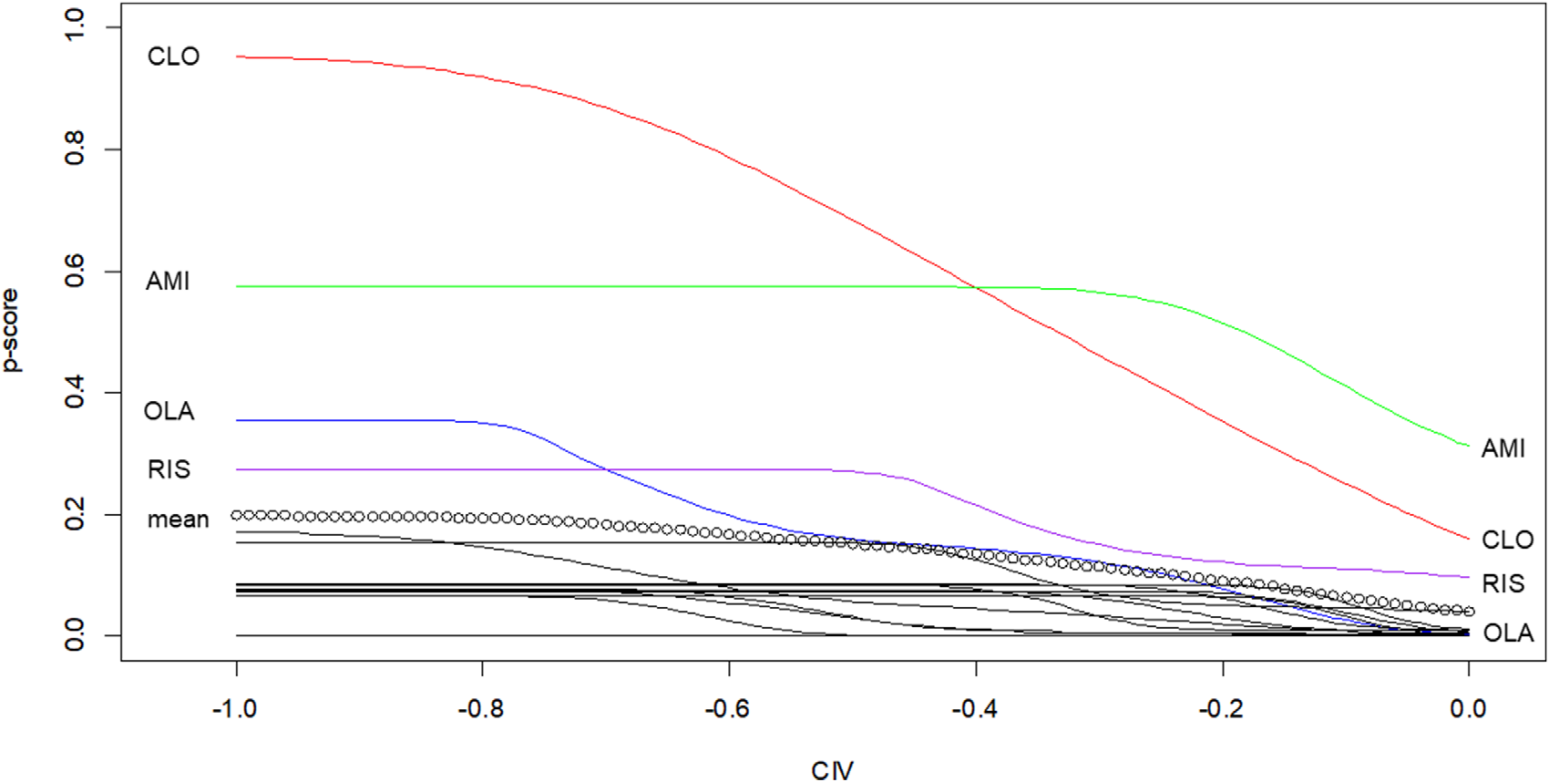
P‐scores for the 16 antipsychotics in terms of efficacy and weight, for various risk (CIV) considerations when we are looking for a benefit of at least 0.2 in the SMD scale for efficacy. We have labeled only the lines with the most effective antipsychotics for illustration purposes. A dotted line is used to show the mean P‐score

In Figure 4, we have set the CIV for efficacy at 0.2 and we estimate P‐scores based on the probability $P(\left(\mu_{i_{O_{1}}}-\mu_{j_{O_{1}}}-0.2<0\right)\cap \left(\mu_{i_{O_{2}}}-\mu_{j_{O_{2}}}-\mathrm{CIV}<0\right))$. This shows how P‐scores fluctuate for various amount of weight gain we are willing to tolerate for a benefit in efficacy equal to 0.2. We considered a correlation of −0.5 between the two outcomes, meaning that the largest the reduction of schizophrenia symptoms, the largest the weight gain. We see that the intersection point of the curves for clozapine and amisulpride is now at CIV=−0.4. We also assumed other values for the correlation between the two outcomes and no changes were observed. Both clozapine and amisulpride single out for the rest of the antipsychotics as these are the only two that have a large probability of getting a difference larger than 0.2 from the rest of the drugs.

## 4 DISCUSSION

Recommending an intervention is a complex issue in which several components such as efficacy, safety, and cost should be taken into account. We argue that ranking metrics should not be treated in isolation but along with the relative effects. Things get more complicated as more outcomes are considered and a benefit–risk assessment is important for making recommendations.

The methods presented here differ for other approaches used in ranking interventions and for benefit–risk assessment. We have extended summary ranking metrics to take into account multiple outcomes and we allow for a benefit–risk assessment using CIVs. Most systematic reviews consider many outcomes but analyze them separately. In this manuscript, we extended the calculation of P‐scores to the case of multiple outcomes. We allow for a joint analysis based on assumptions about the correlation across outcomes. The multi‐outcome version of P‐score refers to the probability that a treatment is better than the remaining treatments in the outcomes considered. We showed how one can extend P‐scores both for one and multiple outcomes so that they allow for certain tradeoffs between benefits and harms. A similar modification to Pbest has been suggested in a Bayesian setting (Brignardello‐Petersen et al., 2018). We think that this extended ranking metric is useful to present at the systematic review level. However, its usefulness and application in the development of recommendations remains to be proven in practice. Treatments with remarkably different safety and efficacy outcomes might produce similar P‐scores and decision‐makers would need to look at each outcome separately in a qualitative manner. Moreover, different stakeholders may weigh outcomes differently (Naci et al., 2014). Summarizing the trade‐offs between benefits and harms is very important and the multi‐outcome version of P‐score provide a summary measure at the NMA level. We showed how one can extend P‐scores both for one and multiple outcomes so that they allow for certain tradeoffs between benefits and harms. A similar modification to Pbest has been suggested in a Bayesian setting (Brignardello‐Petersen et al., 2018).

A visual representation of the benefit–risk assessment will give an overall picture of how ranking fluctuates once we condition on certain CIVs for benefits and harms. Eventually, all P‐scores would converge to zero for increasing gains in efficacy and a visual comparison of the rate of convergence is informative regarding the efficacy of the drug. The visual representation of ranking for certain benefits and risks can be informative and provides a hierarchy of interventions for any set of CIVs. The stakeholder can look at the ranking for the harmful effects (s)he considers acceptable and is willing to exchange for certain benefits.

Ideally, methods presented here would use effect sizes estimated within a multiple outcome NMA so that effects, 95% confidence/credible intervals and correlations among outcomes are estimated in the same setting. This is rarely the case and effects are often estimated in multiple, assumed independent outcome‐specific NMA models. This may theoretically lead to loss in precision and, subsequently, less precise ranking although limited empirical evidence suggests this is unlikely to occur in practice (Trikalinos et al., 2013). Expansion of the method to account for clinically important effects requires estimation of CIVs. However, ideally, the clinician should try to determine the CIVs for the different outcomes—and in particular efficacy and harms—from patients. What we considered in the graphs employed in the motivating example is ranking for a range of CIVs.

Ranking metrics, just like effect estimates, do not (and should not) encompass any information about the risk of bias in the included studies. The credibility of any summary from evidence, be it a relative treatment effect or a treatment hierarchy, need to be evaluated accounting for various evidence characteristics. In an NMA, evaluation of the credibility of relative treatment effects is not straightforward as most studies (that can differ materially in their risk of bias) contribute to the estimation of all treatment effects, either directly or indirectly. Nikolakopoulou et al. (2019) have developed a system for evaluating the Confidence In Network Meta‐Analysis (CINeMA) based on the contribution matrix that shows how much each study contributes to each network estimate and, hence, we can evaluate if an estimate is mainly informed by studies at low risk of bias or not. A natural further step is to extend the CINeMA framework to evaluate an obtained treatment hierarchy as initially described in Salanti et al. (2014).

The approach presented in this manuscript results in many individualized rankings taking multiple outcomes into account. The method is illustrative and reveal graphically how ranking changes for various tradeoffs between benefits and harms. Ranking metrics, when extended along the lines presented in this paper, can reflect clinically important differences on several safety and benefit outcomes. Individualized rankings should not dictate drug prescription but are very informative and a move away from the “one size fits all” standard care of patients to a more personalized one tailored to patient's individual health needs. In practice, other factors not related to the benefit–risk assessment of the interventions such as economic, social, and ethical factors may influence the final decision. Generally, the extended P‐score is a step towards considering multiple outcomes simultaneously and a useful summary measure of an NMA. Their interactions are not always straightforward and prioritizing/weighing outcomes or deciding what deterioration in one outcome could be traded off for certain benefits in another outcome remains a difficult question, both at the individual and population levels (Boers et al., 2010; Yebyo, Aschmann, & Puhan, 2019). As with all quantitative summaries from evidence synthesis, the extended P‐scores run the risk of misinterpretation if users will rely too much on a single measure without considering the actual effect sizes or differences in importance across outcomes.

## CONFLICT OF INTEREST

The authors have declared no conflict of interest.

## Supporting information

The R code can be found in GitHub https://github.com/DimitrisMavridis/RankingNMA/blob/master/extendedP-scores
